# Primary bony non-Hodgkin lymphoma of the cervical spine: a case report

**DOI:** 10.1186/1752-1947-4-35

**Published:** 2010-02-02

**Authors:** Zachary A Smith, Mark F Sedrak, Larry T Khoo

**Affiliations:** 1 Department of Neurosurgery, Ronald Reagan-UCLA Medical Center; Los Angeles, CA 90095, USA

## Abstract

**Introduction:**

Non-Hodgkin lymphoma primarily originating from the bone is exceedingly rare. To our knowledge, this is the first report of primary bone lymphoma presenting with progressive cord compression from an origin in the cervical spine. Herein, we discuss the unusual location in this case, the presenting symptoms, and the management of this disease.

**Case presentation:**

We report on a 23-year-old Caucasian-American man who presented with two months of night sweats, fatigue, parasthesias, and progressive weakness that had progressed to near quadriplegia. Magnetic resonance (MR) imaging demonstrated significant cord compression seen primarily at C7. Surgical management, with corpectomy and dorsal segmental fusion, in combination with adjuvant chemotherapy and radiation therapy, halted the progression of the primary disease and preserved neurological function. Histological analysis demonstrated an aggressive anaplastic large cell lymphoma.

**Conclusion:**

Isolated primary bony lymphoma of the spine is exceedingly rare. As in our case, the initial symptoms may be the result of progressive cervical cord compression. Anterior corpectomy with posterolateral decompression and fusion succeeded in preventing progressive neurologic decline and maintaining quality of life. The reader should be aware of the unique presentation of this disease and that surgical management is a successful treatment strategy.

## Introduction

Primary vertebral lymphoma presenting without sites of systemic involvement is exceedingly rare. Bone is the primary origin of the disease in only 1% of non-Hodgkin lymphomas [1]. Further, primary vertebral locations account for only 1.7% of all primary bone lymphomas, and the vast majority occur in short bones, with a predilection for the scapula, ileum, femur, and tibia [1,2]. Bony and epidural non-Hodgkin lymphoma has been described in small case series when it has involved the thoracic and lumbar spine [3,4]. However, to our knowledge, the presentation of primary bone lymphoma of the cervical spine is unique to the clinical literature.

Moreover, when it occurs in a primary vertebral location, the tumor can often grow insidiously and reach considerable size and clinical consequence prior to diagnosis. When this occurs, the symptoms may be rapidly progressive, and surgical decompression and stabilization may be required to preserve function and maintain quality of life. This presents a successful and requisite strategy in the management of this unique clinical presentation.

## Case presentation

**Figure 1:**
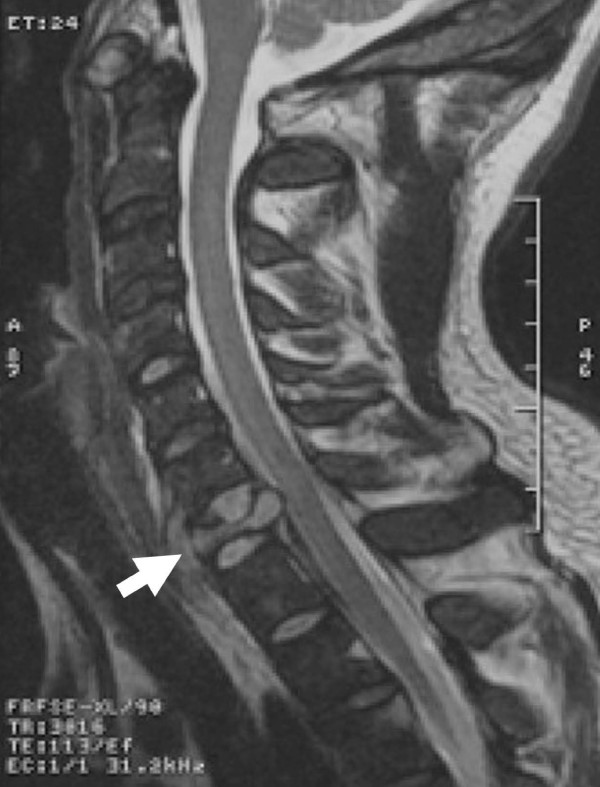
**Pre-operative imaging with MRI demonstrating destruction and collapse of the C7 body (A)**. Compression of the spinal cord in the epidural space is shown with distortion of the cord at this level.

A 23-year-old Caucasian-American man presented to our medical center with type-B symptoms, including two months of fever, night sweats, and progressive weight loss. The patient had a negative HIV status and normal CD4 count. Additionally, he had left upper extremity weakness and paresthesias. The initial workup was initiated at an outside medical center where the patient presented with persistent left shoulder pain refractory to medical pain management. He then developed weakness of the bilateral upper extremities and decreased sensation to light touch. The symptoms worsened during his hospital course, and he became bed-bound from generalized weakness and fatigue. Low-volume lumbar puncture was undertaken after negative imaging of the head. This revealed a normal protein count and only 13 white blood cells. However, his peripheral white blood cell count was 15.5 × 10^9^/L, and his alkaline phosphatase was significantly elevated. An MR was obtained which demonstrated cervical cord compression at the C7 level with near complete bilateral foraminal stenosis (Figure 1). There were striking changes on MR with virtually complete destruction, collapse and replacement of the C7 vertebra by a soft tissue mass with an associated epidural component and resultant narrowing of the spinal canal. In addition, numerous multicentric osseous lesions were also observed in the adjacent cervical levels that were hypointense on T1, hyperintense on T2, and bright on gadolinium-enhanced T1 sequences. A bone scan demonstrated multiple areas of increased radiotracer uptake in the lower cervical spine, centered at C7. A lymph node biopsy of a metachronous associated parasternal node yielded histopathological characteristics consistent with an anaplastic large T-cell lymphoma. However, CT scans of chest, abdomen, and pelvis, as well as full body PET and bone scans failed to show additional evidence of disease.

At time of transfer to our facility, the patient had near full strength of the right side, but he was weak against resistance on his left. On the third day after transfer it was noticed that his left side had acutely progressed to only a flicker of movement in the hands, and his right-sided strength had also significantly diminished. Rectal tone was found to be diminished. Given the known compression on MR imaging, urgent surgical decompression and fusion was elected to prevent progressive neurological decline.

The patient underwent a single-staged anterior and posterior procedure with anterior C7 corpectomy and decompression, cage placement, and C6-T1 anterior plate stabilization. This was followed by a posterior C5-T2 lateral mass screw-rod stabilization and posterolateral arthrodesis. At the time of operation, there was significant prevertebral swelling in the fascia anterior to C7 and a clear destructive and invasive process at this level. The bone was thoroughly invested with tumor, and the anatomy of the vertebral body was distorted by erosion from the malignancy. The dura of the cord was noted to be hemorrhagic and severely discolored in many areas where it had been encased and compressed by the tumoral mass that had both a lobular and en-plaque type growth pattern seen. Of prominent note, the C7 and T1 vertebral bone quality was significantly compromised and there was postural instability at bilateral C4/C5 and C5/C6 facets. The extent of bony destruction had exceeded pre-operative expectations and prompted both additional posterior fusion as well as hardware placement a level above and below anterior reconstruction.

Histological specimen analysis was consistent with a highly aggressive anaplastic large cell lymphoma. The tumor was CD30+ and CD5+ and negative for other markers, suggestive of a T-cell phenotype. Immunohistochemistry was positive for EMA and ALK-1. Microscopic examination demonstrated extensive infiltration of large atypical cells with bizarre nuclei, prominent nucleoli, and evidence abundant mitotic figures and apoptotic bodies. The histology demonstrated extensive infiltration of the bone from the C7 vertebral body, and the disease process at this site was significantly more involved than the adjacent soft-tissue.

**Figure 2:**
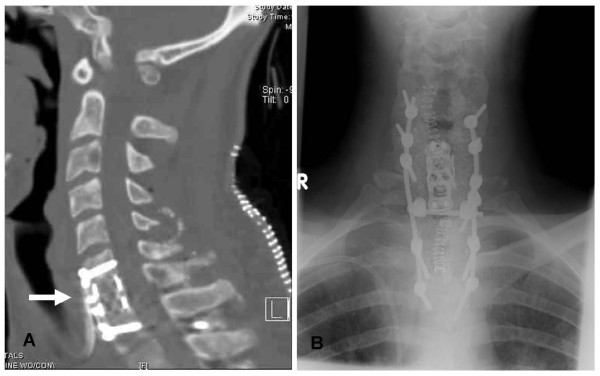
**Post-operative CT imaging demonstrates the placement of C7 corpectomy cage (A); Plain X-ray showing ventral anterior plate and corpectomy cage and dorsal hardware, with lateral mass screws, in place (B)**.

Post-operatively, early mobilization while wearing a cervical collar was encouraged. Collar immobilization was continued in the weeks following surgery as he underwent chemotherapy. He had a near-complete return of strength in his extremities without residual post-operative sensory deficits. Post-operative imaging revealed proper alignment and stability of cage placement with excellent restoration of the spinal canal patency and subarachnoid space (Figure 2). Following a short period of post-operative recovery, the patient was started on an initial cycle of a CHOP chemotherapy regimen (Per cycle, Day 1: cyclophosphamide 750 mg/m^2 ^IV, doxorubicin 50 mg/m^2 ^IV, vincristine 1.4 mg/m^2 ^IV; Day 1-5: prednisone 100 mg po) to treat his adjacent bony spinal lesions. Six cycles of 21-day length were planned. Repeat imaging of body with CT imaging as well as with nuclear imaging modalities (PET and MR-spectroscopy) failed to demonstrate extra-spinal disease at 14 weeks post-operative.

In the immediate months following surgery the patient regained near full strength. Three weeks post-op, he was discharged from the hospital with home physical therapy. At time of last follow-up, one year after the operation, the patient continued to have a stable functional status.

## Discussion

Primary bony involvement at the time of presentation is uncommon with non-Hodgkin lymphoma. When symptoms are advanced, it may be difficult to determine if the source is bony in origin or secondary to spread from a distant site. An accepted definition of primary bony lymphoma is bone involvement, without evidence of disseminated disease [5]. A more thorough definition is that of a single lesion that persists for longer than six months without evidence of systemic involvement [1,6]. This excludes the possibility of an undetected primary source of disease. This form of non-Hodgkin lymphoma is especially rare. It accounts for only 1% of all cases, and of these, primary vertebral locations account for only 1.7% of primary bone lymphomas [1,2]. In our case, the patient presented with cervical spinal compression from an osseous lesion consistent with an origin at the C7 vertebral body. This was supported by pre-operative imaging, inter-operative findings, and pathological analysis. There was no source of disseminated or extraspinal disease at presentation, nor at six months after initial diagnosis. To our knowledge, this case is the first report of such a clinical presentation.

Primary bone lymphoma is often characterized by osteolytic defects. However, this may be overlooked on plain X-rays [2]. Local pain and type B symptoms, including generalized fatigue, night sweats, and weight loss are common [7]. These are often non-specific findings, but prompt further diagnostic work-up [2,8]. Tissue diagnosis with biopsy of an adjacent lymph node or directly from the involved bone forms the foundation of the diagnosis. When diagnosed through biopsy, high grade tumors are rare, and the most common grade identified is intermediate, followed by low grade lesions [4,9].

When lymphoma involves the spine, it is more commonly found isolated to the epidural space and as a result of secondary spread [1,10]. Tumor location within the epidural space can vary, but there is a proclivity for the thoracic levels [4,11]. Small case series with spinal epidural origin have been reported [3,4]. These were primarily thoracolumbar in location and thought to result from an epidural, rather than bony origin. Although controversial, it is felt that primary epidural lymphomas may derive from normal lymphoid tissue in the epidural space [12]. However, the possibility of seeding from adjacent lymph nodes cannot be excluded [13].

Regardless of the spinal level or site of origin, patients with spinal lymphoma are at risk for neurological deterioration. The resultant clinical findings from any compressive spinal epidural mass may be pain, motor weakness, sensory deficits, and potential bowel or bladder dysfunction. If left untreated, this may progress to paralysis. The treatment of these lesions is often multifactorial, and includes surgery, radiotherapy, and chemotherapy [14]. A discussion of the pros and cons of these treatment options is beyond the scope of this report. However, in cases of acute neurological decline, we support early surgical decompression. The management and outcome of this case demonstrates a successful means of managing this clinical presentation.

## Conclusions

This case brings attention to a unique presentation of non-Hodgkin's lymphoma in the cervical spine. Non-Hodgkin's lymphoma rarely originates from bone, and even more infrequently arises from the vertebral bodies. We describe an aggressive lymphoma that arose from the C7 vertebral body. In this case, we chose to surgically decompress and fuse the patient, given the speed of our patient's neurological decline and the extent of bony destruction. The presented case provides an example of a unique presentation of disease and an example of successful management.

## Abbreviations

MR: Magnetic resonance; EMA: Anti-endomysial antibody; ALK: Anaplastic lymphoma kinase; PET: Positron emission tomography; HIV: Human immunodeficiency virus; EMA: Epithelial membrane antigen; ALK: Anaplastic lymphoma kinase; PET: Positron emission tomography.

## Consent

Written informed consent was obtained from the patient for publication of this case report and any accompanying images. A copy of the written consent is available for review by the Editor-in-Chief of this journal.

## Competing interests

The authors declare that they have no competing interests.

## Authors' contributions

ZAS contributed to the final approval, data collection, interpretation of the data and literature, revision, and drafting of the manuscript. MFS contributed to revision of the manuscript and data collection. LTK contributed to the final approval and revision of manuscript.
